# Clinical characteristics of myelin-oligodendrocyte glycoprotein antibody-positive pediatric autoimmune encephalitis without demyelination: A case series

**DOI:** 10.3389/fimmu.2022.1050688

**Published:** 2022-12-01

**Authors:** Xiaojie Song, Jiannan Ma

**Affiliations:** Department of Neurology, Children’s Hospital of Chongqing Medical University, National Clinical Research Center for Child Health and Disorders, Ministry of Education Key Laboratory of Child Development and Disorders, Chongqing Key Laboratory of Pediatrics, Chongqing, China

**Keywords:** autoimmune encephalitis, myelin-oligodendrocyte glycoprotein antibody, MOG antibody-associated diseases, brain magnetic resonance imaging, children

## Abstract

**Purpose:**

To facilitate the identification of myelin-oligodendrocyte glycoprotein (MOG) antibody-associated diseases in pediatric autoimmune encephalitis without demyelination, we explored the clinical characteristics of patients having MOG antibody-positive pediatric autoimmune encephalitis without demyelination in Children’s Hospital of Chongqing Medical University, China.

**Methods:**

We reviewed patients’ medical records from January 2019 to June 2022 and retrospectively analyzed clinical manifestations, brain magnetic resonance imaging (MRI) findings, laboratory findings, treatments and outcomes of children with autoimmune encephalitis who tested positive for MOG antibodies in serum but for whom demyelination was not detected on MRI.

**Results:**

Eighteen patients (6 boys, 12 girls; median age: 103.2 (range: 36–160) months) were included: 15 tested positive for MOG antibodies in both serum and cerebrospinal fluid (CSF); three tested positive only in serum. The most common clinical symptoms were altered mental status (18/18), fever (16/18), headache (14/18), seizures (6/18) and focal neurologic deficits (5/18). All patients had CSF pleocytosis (median count: 74/µL, range: 14–380/µL); five patients had elevated CSF protein levels (median: 0.85 g/L, range: 0.53–1.48 g/L) simultaneously. CSF glucose levels were normal in all patients. Abnormal electroencephalogram (EEG) results were found in 12 patients: generalized or focal slowing (9/12), focal epileptic discharges (2/12), and generalized slowing and focal epileptic discharges (1/12). Twelve of the 18 patients showed hyperintense T2-weighted lesions on brain MRI in the cortex (6), basal ganglia (5), thalamus (3), cerebellum (4), and brainstem (2). All patients received immunotherapy and had favorable outcomes at discharge (modified Rankin scale score: <2). Three children relapsed once; however, all children had good outcomes at the last follow-up.

**Conclusion:**

MOG antibody-positive pediatric autoimmune encephalitis without demyelination is mainly characterized by prolonged fever, altered mental status, headache, mild-to-moderate increase in cell count in the CSF, and normal or abnormal brain MRI, which may involve any part outside the white matter without specificity. All patients with MOG antibody-positive pediatric autoimmune encephalitis without demyelination had favorable outcomes after immunotherapy, while a few patients relapsed once.

## Introduction

Myelin-oligodendrocyte glycoprotein (MOG) is an important component of myelin in the central nervous system (CNS) and is expressed at the external surface of myelin and the plasma membranes of oligodendrocytes (1). The antibody to MOG is involved in various demyelinating disorders of CNS, including acute disseminated encephalomyelitis (ADEM), optic neuritis (ON), myelitis, and neuromyelitis optic spectrum disorder (NMOSD) (2, 3). Multinational studies have identified serum anti-MOG antibody positivity in approximately 40% of children with acute demyelinating syndrome (1, 4).

In recent years, the anti-MOG antibody has been reported to be associated with various types of autoimmune encephalitis (AE) without demyelination, such as cortical encephalitis, brainstem encephalitis, and even AE with normal findings on brain magnetic resonance imaging (MRI) (5–10). As more and more MOG antibody-associated phenotypes emerged, the concept of MOG antibody-associated diseases (MOGAD) was proposed (1). However, relevant reports about MOG antibody-associated AE without demyelination were usually case reports, and there were few pertinent studies with large samples, especially including Asian children. Therefore, in this study, we assessed the cases of 18 children having MOG antibody-positive AE without demyelination in a single center in southwest China.

## Methods

### Patients

Medical records of children who were hospitalized at Children’s Hospital of Chongqing Medical University between January 2019 and June 2022 were reviewed. Those who met the following criteria were included in the study: (i) patients who tested positive for MOG antibodies in the serum, (ii) patients who fulfilled the criteria for possible AE (11), (iii) patients whose brain MRI revealed no demyelination (i.e. no involvement of the cerebral white matter), and (iv) patients for whom alternative causes (e.g., intracranial infections or autoimmune diseases) could be reasonably excluded.

Patients’ clinical features, MRI findings, laboratory findings, treatment, and outcomes were analyzed retrospectively. After discharge, all patients were followed up by outpatient visits. Severity at onset and outcome at the last clinical evaluation were evaluated with the modified Rankin scale (mRS) (12). This study was approved by the ethics committee of Children’s Hospital of Chongqing Medical University. Written informed consent was obtained from the children’s parents or legal guardians.

### Assays for MOG antibodies

MOG antibodies in serum and CSF were tested by using a fixed cell-based assay involving HEK293 cells co-transfected with human MOG and pcDNA3.1-EGFP.

## Results

### Clinical findings

Eighteen patients who met the inclusion criteria were included: fifteen and three patients tested positive for MOG antibodies in both serum and CSF and in serum only, respectively. The demographic and clinical characteristics of the 18 patients are shown in **Table 1**. The median patient age was 103.2 months (range: 36–160 months), and 6 patients were male (33.3%). All patients had no previous history of disease in the central nervous system.

**Table 1:** Detailed demographic and clinical characteristics of 18 patients having MOG antibody-positive pediatric autoimmune encephalitis without demyelination.

Patients No. sex/age (months)	Clinical presentation	Duration of fever (days)	Brain MRI findings	EEG	Peripheral white blood cell count, ×10^9^/L; neutrophil count; CRP level (mg/L); PCT level (ng/mL)	CSFWBC count,/μL; protein level, g/L;glucose level, mmol/L	Treatment before diagnosis	Time to diagnosis (days)	Immunotherapy	mRS on admission; worst mRS; mRS at discharge	Length of stay (days)	Relapse, time to relapse (months)	mRS at the last follow-up, follow-up duration (months)
1.F/122	Fever, headaches, lethargy	21	Normal	ND	31.23; N 0.89; CRP<8; PCT 0.09	126;1.11;2.52	Cephalosporin + vancomycin	26	IVMP OP	3; 3; 1	17	None	0/22
2.M/36	Fever, dizziness, irritability, lethargy	15	Normal	Generalized slowing	33.61; N 0.73; CRP<8; PCT <0.05	240;0.85;2.88	Vancomycin + meropenem	21	IVMP OP	2; 3; 0	28	None	0/17
3.F/132	Fever, headaches, seizures, somnolence,cognitive deficit	5	Normal	Generalized slowing	13.2, N 0.75, CRP<8, PCT <0.05	75;0.33;4.45	Acyclovir	9	IVIG IVMP OP	3; 3; 1	16	Y/6	1/16
4.F/108	Fever, headaches, lethargy	15	Normal	ND	19.55, N 0.81, CRP<8, PCT <0.05	362;1.48;2.7	Cephalosporin+vancomycin	14	IVIG	2; 3; 0	22	None	0/15
5.F/123	Fever, headaches, seizures, somnolence	13	Normal	Generalized slowing	10.83, N 0.76, CRP<8, PCT <0.05	28;0.36;2.63	Cephalosporin	70	OP	2; 2; 0	11	None	0/23
6.M/160	Fever, headaches, somnolence	8	Normal	Right frontal SWs	18.98, N 0.86, CRP 26; PCT <0.05	100;0.31;2.37	Cephalosporin	8	IVIG IVMP OP	3; 3; 1	13	None	0/8
7.M/52	Fever, headaches, somnolence, dysarthria	4	Bilateral T2-hyperintense patchy lesions in the cerebellum	Generalized slowing	29.14, N 0.8, CRP<8, PCT <0.05	72;0.42;2.98	Cephalosporin + acyclovir	11	IVIG	3; 3; 1	14	None	0/15
8.M/60	Fever, headaches, somnolence	11	Bilateral T2-hyperintense patchy lesions in the basal ganglia, thalamus, and cerebellum	Generalized slowing	26.75, N0.89, CRP<8, PCT 0.06	84;0.53;2.8	Meropenem	17	IVIG IVMP OP	3; 3; 1	21	None	0/14
9.F/72	Fever, headaches, somnolence	5	T2-hyperintense lesions in the right basal ganglia	Normal	16.13, N 0.83, CRP<8, PCT 0.1001	53;<0.1;2.97	Acyclovir	41	IVIG OP	2; 2; 0	23	None	0/17
10.F/132	Fever, headaches, seizures, hemiparesis, somnolence	6	T2-hyperintense lesions in the right frontal cortex	Generalized slowing, bilateral temporal SWs	21.54, N 0.84, CRP<8, PCT <0.05	22;0.28;2.73	Acyclovir	15	IVIG IVMP OP	3; 3; 1	21	None	1/16
11.F/156	Seizures, somnolence	0	T2-hyperintense lesions in the center insular lobe cortex	ND	24.03, N 0.86, CRP<8, PCT <0.05	18;0.29;3.25	Cephalosporin	7	IVIG IVMP OP	3; 3; 0	11	Y/19	0/35
12.F/84	Fever, headaches, somnolence, blepharoptosis, ataxia, dysarthria	7	Bilateral T2-hyperintense patchy lesions in the basal ganglia, right cerebellum, and brainstem	Normal	7.41, N 0.62, CRP<8, PCT <0.05	19;0.29;3.14	ND	19	IVIG IVMP OP	4; 4; 1	12	None	1/35
13.F/144	Fever, headaches, somnolence	19	T2-hyperintense patchy lesions in the center basal ganglia	Generalized slowing	21.02, N 0.86, CRP 12, PCT <0.05	82;0.44;2.79	Cephalosporin	25	IVIG IVMP OP	2; 3; 0	25	None	1/36
14.F/48	Fever, headaches, somnolence	24	Bilateral T2-hyperintense lesions in the thalamus and right frontal cortex	Generalized slowing	22.59, N 0.80, CRP<8, PCT <0.05	36;0.27;3.02	Cephalosporin	29	IVIG IVMP OP	2; 3; 0	13	None	1/14
15.F/65	Fever, headaches, somnolence	7	Bilateral T2-hyperintense lesions in the thalamus, brainstem, and center temporal	Generalized slowing	14.55, N 0.64, CRP<8, PCT <0.05	30;0.19;2.74	Acyclovir	11	IVIG IVMP OP	2; 3; 0	14	None	1/13
16.M/102	Fever, headaches, seizures, somnolence	14	T2-hyperintense lesions in the center temporal and insular regions and basal ganglia	Slowing in the center hemisphere	13.27, N 0.71, CRP<8, PCT 0.194	380;0.59;2.65	Cephalosporin	13	IVIG IVMP OP	3; 3; 1	22	Y/26	1/39
17.M/130	Fever, seizures, somnolence	0	T2-hyperintense patchy lesions in the right frontal cortex	Right frontal SWs	12.45, N 0.87, CRP<8, PCT <0.05	14;0.13;3.21	Acyclovir	9	IVIG OP	2; 3; 0	14	None	1/17
18.F/131	Fever, dizziness, lethargy, ataxia, strabismus	10	T2-hyperintense patchy lesions in the center parietal cortex and right cerebellum	Normal	13.54, N 0.81, CRP<8, PCT <0.05	200;0.35;3.8	ND	19	IVIG IVMP OP	4; 4; 1	27	None	1/37

CSF, cerebrospinal fluid; CRP, C-reactive protein; F, female; IVMP, intravenous methylprednisolone; IVIG, intravenous immunoglobulin; M, male; MOG, myelin oligodendrocyte glycoprotein; mRss, modified Rankin scale score; MRI, magnetic resonance imaging; N, neutrophil; ND, not done; OP, oral prednisone; PCT, procalcitonin; SWs, spike waves; WBC, white blood cell.

The most common clinical symptoms were altered mental status (18/18, lethargy in 4, somnolence in 14); fever (16/18) lasting for 4–24 days (median: 10.5 days); headaches (14/18); seizures (6/18); and focal neurologic deficits (5/18), including dysarthria (2/18), ataxia (2/18), extraocular muscle disorder (2/18), and hemiparesis (1/18). All the patients had no movement disorder, psychiatric/behavioural symptoms, or dysautonomia.

### Laboratory findings

Seventeen in 18 patients had leukocytosis in peripheral blood (median: 19.55 × 10^9^ cells/L, range: 10.83–33.61 × 10^9^ cells/L, normal value: 4–10 × 10^9^ cells/L). C-reaction protein (CRP) and procalcitonin (PCT) were mildly elevated in two patients (range: 12–26 mg/L, normal value: <8 mg/L) and 4 patients (range: 0.06–0.194 ng/mL, normal value: <0.05 ng/mL), respectively. All patients had CSF pleocytosis (median: 74/µL, range: 14–380/µL, normal value: ≤5/µL); five patients had elevated CSF protein levels (median: 0.85 g/L, range: 0.53–1.48 g/L, normal value: ≤0.45 g/L). All patients had normal CSF glucose levels. Bacterial culture and polymerase chain reaction for herpes simplex virus (HSV) and Epstein-Barr virus (EBV) in the CSF were negative for all patients.

### Electroencephalogram findings

Electroencephalogram (EEG) was performed in 15 patients, revealing generalized or focal slowing in nine children, focal epileptic discharges in two children, and generalized slowing combined with focal epileptic discharges in one child, and normal EEG in three patients.

### MRI findings

**Figure 1:**
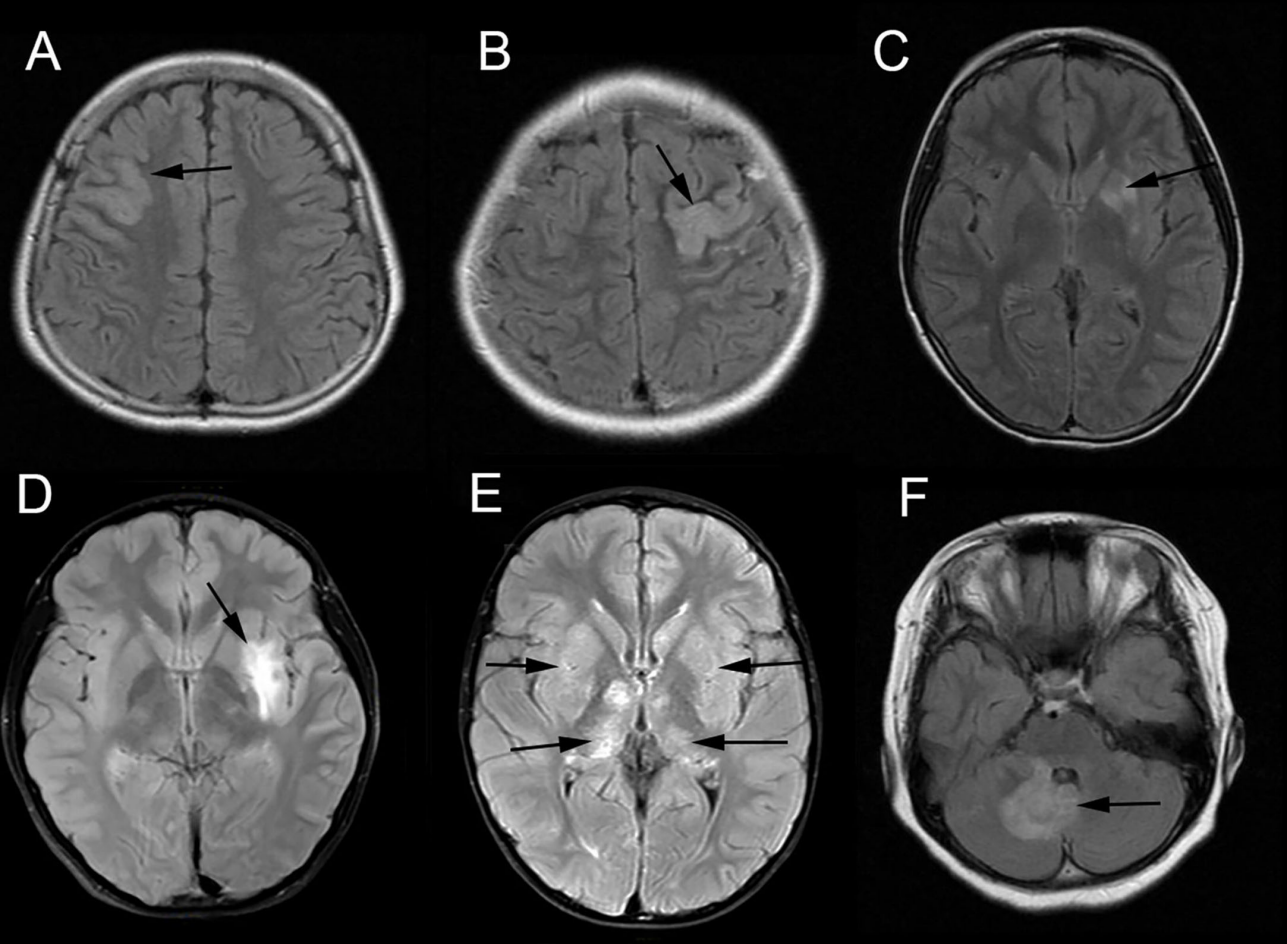
Brain magnetic resonance imaging (MRI) of patients having MOG antibody-positive pediatric autoimmune encephalitis without demyelination. The abnormal hyperintensity lesions on axial T2-weighted fluid-attenuated inversion recovery (T2-FLAIR) image were observed in the right frontal cortex (**A**, Patient 17), left parietal cortex (**B**, Patient 18), left basal ganglia (**C**, Patient 13), left insular cortex and basal ganglia (**D**, Patient 16), bilateral basal ganglia and thalamus (**E**, Patient 8) and right cerebellum (**F**, Patient 18).

Brain MRI findings were showed in **Table 1**. Brain MRIs were performed for all 18 patients. Hyperintense lesions on T2-weighted images were noted in 12 patients (**Figures 1 A–F**), while the findings were negative in six patients. The distribution of lesions was unilateral in six patients and bilateral in six patients. The abnormal T2-hyperintense lesions were located in the cortex (n = 6, including parietal [2/6], temporal [3/6], frontal [1/6], and insular [2/6] regions), basal ganglia (n = 5), thalamus (n = 3), cerebellum (n = 4), and brainstem (n = 2). Brain MRI showed isolated lesions in six patients (isolated cortex [n = 3], isolated cerebellum [n = 1], and isolated basal ganglia [n = 2]), combined lesions in six other patients (cortex combined with basal ganglia or thalamus [n = 3, one involved the brainstem], cortex combined with the cerebellum [n = 1], basal ganglia combined with the thalamus and cerebellum [n = 1], and basal ganglia combined with the brainstem and cerebellum [n = 1]). Gadolinium -enhanced brain MRI was performed in 17 patients (except patient 5), 16 of whom had no enhancement, and only one patient (patient 8) had slightly enhancement in the right cerebellum. Diffusion Weighted Imaging (DWI) was performed in all patients, but diffusion restriction was observed in none of them.

### Treatment and outcomes at discharge

The treatment and outcomes at discharge in the 18 patients are shown in **Table 1**. Before the diagnosis of MOGAD, anti-infection therapy was administered empirically due to suspected intracranial infections. Ten patients received antibiotic therapy, five patients received acyclovir, and one patient received both antibiotics and acyclovir. However, no patient responded to anti-infection therapy. The median time to diagnosis after onset was 14.5 days (range: 7–70 days) in all patients. All patients were treated with immunotherapy after MOGAD was confirmed. In our center, the general immunotherapy program for patients with MOG antibody-positive pediatric autoimmune encephalitis without demyelination is intravenous immunoglobulin (IVIG, 400 mg/kg/day for 5 days) followed by sequential administration of intravenous methylprednisolone (IVMP, 15–20 mg/kg/day for 3–5 days) and oral prednisone (OP, 1.5–2.0 mg/kg/day). If the condition is significantly improved after the use of IVIG, the IVMP can be omitted. However, in clinic, sometimes patients’ parents would refuse to use IVIG due to economic reason or corticosteroids in consideration of their side effect. So, in our study, 15 of the 18 patients were treated with IVIG, of whom 11 were subsequently treated with IVMP followed by OP, 2 were followed by OP only, and 2 without corticosteroids. Two of the 18 patients were treated with IVMP followed by OP, and one patient was treated with OP alone. For the patients received OP, the drug was tapered off within 3-6 months. The modified Rankin scale (mRS) score decreased in all patients after immunotherapy, and all patients had favorable outcomes at discharge (mRS score < 2). The median length of stay was 16.5 days (range: 11–28 days) in all patients.

### Follow-up

After discharge, all patients were followed up by outpatient visits. The follow-up duration was between 8 and 39 months, with the average being 21.67 months. During follow-up, three patients relapsed once (patient 3, patient 11, patient 16). The patient 3 presented with seizure once 6 months after discharge. Her repeated brain MRI showed T2-hyperintense lesions in the right thalamus after relapsed, EEG had no positive finding, and MOG antibodies in both serum and CSF were positive. The patient 11 suffered seizure once 9 months after discharge with positive MOG antibodies in both serum and CSF, but she had normal EEG and brain MRI findings. The patient 16 presented with optic neuritis in the right eye with MOG antibodies positive in serum 26 months after discharge. His checkerboard visual evoked potential showed increased latency time and decreased amplitude of p100 wave for the right eye, but his brain and optic nerve MRI finding were normal. All the three patients experienced symptom relief after receiving IVMP followed by OP. Further, those three patients were also treated with immunosuppressive treatments (such as mycophenolate mofetil (MMF) or azathioprine) to prevent relapses. All patients had favorable outcomes at the last follow-up (mRS score < 2).

## Discussion

In this study, we evaluated the clinical characteristics of 18 pediatric patients having autoimmune encephalitis with positive MOG antibodies in the serum but without demyelination on brain MRI. To our knowledge, this is the first study on Asian children to specifically report the clinical characteristics of the spectrum of MOG antibody-positive pediatric autoimmune encephalitis without demyelination.

In a prospective study, Armangue et al. (13) showed that among 296 children with definite or possible encephalitis other than ADEM, MOG antibodies were more common than NMDAR antibodies (22 [7.4%] VS 14 [4.7%]), and surpassed all neuronal antibodies combined. During the same period (January 2019 to June 2022), 88 patients were diagnosed with anti-NMDAR encephalitis in our center. Not all patients with suspected autoimmune encephalitis were tested for anti-MOG antibodies in our retrospective study. Therefore, whether the anti-MOG antibody is the most common antibody in Asian patients having autoimmune encephalitis without demyelination needs to be confirmed in more prospective studies.

In this study, the main clinical manifestations in patients having MOG antibody-positive autoimmune encephalitis without demyelination were altered mental status, prolonged fever, and headaches. However, these clinical manifestations are non-specific. In contrast, anti-NMDAR encephalitis, which was considered the most common type of autoimmune encephalitis in children, is characterized by behavioral problems and/or movement disorders (11, 14).

CSF tests revealed abnormal findings in all patients in our study. All patients had CSF pleocytosis, and a few (5/18) had elevated CSF protein levels. No patient had an abnormal glucose level. In the study by Armangue et al. (13), all encephalitis patients with anti-MOG antibody positivity other than ADEM had varying degrees of CSF leukocytosis. However, none of them showed elevated protein levels.

Interestingly, almost all patients (17/18) had leukocytosis in peripheral blood, with half of them having a cell count higher than 20 × 10^9^/L. Based on our experience, leukocytosis in peripheral blood is rare in other types of autoimmune encephalitis (e.g., anti-NMDAR encephalitis). Therefore, many of our patients were suspected of having bacterial meningitis and administered antibiotics for a long time before the diagnosis of MOGAD, and the longest time to diagnosis was approximately 70 days. However, the following findings are especially important. (1) No patient showed significantly increased levels of the inflammatory indicators CRP and PCT. In fact, most patients had normal levels of these inflammatory indicators. (2) CSF glucose levels were normal in all patients. (3) Finally, no patient responded to antibiotic therapy.

The brain MRI of six patients was normal. Armangue et al. (13) have reported two cases of encephalitis with normal brain MRI findings and anti-MOG antibody positivity. However, they did not describe the specific clinical characteristics of these two patients. In our study, the main clinical manifestations of the aforementioned six patients were fever, a slightly altered mental status, and headaches (5/6); pleocytosis in the CSF; and normal CSF glucose levels. Given these clinical manifestations, the condition needs to be differentiated from viral meningitis and systemic diseases [e.g., systemic lupus erythematosus (SLE), Kawasaki disease] with meningeal involvement. However, five of these patients had a fever that lasted for more than 1 week (up to 21 days) before immunotherapy, which is not consistent with viral meningitis because viral meningitis is self-limited (duration is usually less than 1 week). None of these 6 patients had rashes, lymphadenopathy, other common manifestations of systemic diseases, and elevated levels of the inflammatory indicator CRP. Therefore, systemic disease was not considered. The brain MRI of the other 12 patients was abnormal. However, the lesions had no specificity and involved the cerebral cortex, basal ganglia, thalamus, cerebellum, or brainstem. Such lesions may be isolated (isolated cortex, cerebellum, or basal ganglia) or combined (e.g., involving the cortex combined with basal ganglia or thalamus or basal ganglia combined with thalamus). Therefore, differentiating them from the lesions associated with viral encephalitis based on brain imaging alone may be difficult. Hence, we tested CSF for HSV and EBV to exclude HSV and EBV encephalitis). Most patients were empirically treated with anti-infection therapy before the diagnosis of MOGAD. Therefore, the detection of MOG antibodies was crucial for confirming the diagnosis in these patients.

All of our patients had a good response to intravenous immunotherapy and had a good prognosis at discharge (mRS score < 2). This finding was consistent with those reported for most patients with MOG antibody-positive demyelinating syndromes. It’s worth mentioning that the immunotherapy strategy for our patients differs slightly from available consensus recommendations of pediatric MOGAD due to the previous suspicions of intracranial infections in these patients (15). However, in our study, three patients treated with the corticosteroids alone also experienced remission, which suggested the IVIG treatment might not be necessary for some of our patients. During follow-up, three patients relapsed; the rate was comparable to that of MOG antibody-positive demyelinating syndromes (16.7% VS 33%) (13). These three patients improved rapidly after re-administration of intravenous immunotherapy and had a good prognosis at the final follow-up.

The pathological mechanism underlying the generation of anti-MOG antibodies in pediatric autoimmune encephalitis without demyelination is unknown. Theoretically, the anti-MOG antibody, as a kind of extracellular antigen located on the surface of myelin, should only be associated with inflammatory demyelinating diseases of the white matter in the central nervous system (such as ADEM and ON) (16). Some previous reports have described cortical encephalitis and meningoencephalitis without demyelination and positive results for anti-MOG antibodies (6–8, 11). Therefore, for encephalitis without demyelination, the anti-MOG antibody itself may not be directly associated with the encephalitis and another unknown autoimmune disorder coexisting with anti-MOG antibody positivity may be responsible for the encephalitis. In addition, MOG is not only an important component of the myelin sheath but also a type of myelin inhibitor of axonal regrowth (17). Whether it is another possible pathological mechanism that even though anti-MOG antibodies do not lead to demyelination, may damage the structures and functions of neurons through destroying the function of MOG as a kind of inhibitor of axonal regrowth. Nevertheless, this is only a hypothesis, and more animal and clinical research is needed to prove it.

Our study had some limitations. First, we did not detect neuronal surface antibodies (such as anti-NMDAR antibodies) in all patients. However, we did not think our patients had coexisting neuronal surface antibodies since pediatric autoimmune encephalitis with neuronal surface antibodies mainly manifests as behavioral problems and/or movement disorders. Second, the follow-up period was relatively short for some patients. Hence, determination of the true recurrence rate of MOG antibody-positive pediatric autoimmune encephalitis without demyelination will require a longer follow-up. Finally, our study was a single-center retrospective study with a small number of cases. Hence, more prospective multicenter studies with larger samples are needed in the future.

## Conclusion

For patients with suspected encephalitis manifesting as altered mental status, prolonged fever, headaches, and leukocytosis in the CSF, anti-MOG antibody-associated diseases should be concerned and the anti-MOG antibody test should be performed early to avoid a delayed diagnosis, even for those whose brain MRI is normal or shows no demyelination lesions. Our study shows that patients having MOG antibody-positive pediatric autoimmune encephalitis without demyelination usually have favorable outcomes after immunotherapy, while a few may relapse.

## Data availability statement

The raw data supporting the conclusions of this article will be made available by the authors, without undue reservation.

## Ethics statement

The studies involving human participants were reviewed and approved by the Ethics Committee of Children’s Hospital of Chongqing Medical University. Written informed consent to participate in this study was provided by the participants’ legal guardian/next of kin.

## Author contributions

XS collected clinical records, analyzed and interpreted the data, drafted and revised the manuscript. JM designed and conceptualized the study and revised the manuscript. Both authors contributed to the article and approved the submitted version.

## Conflict of interest

The authors declare that the research was conducted in the absence of any commercial or financial relationships that could be construed as a potential conflict of interest.

## Publisher’s note

All claims expressed in this article are solely those of the authors and do not necessarily represent those of their affiliated organizations, or those of the publisher, the editors and the reviewers. Any product that may be evaluated in this article, or claim that may be made by its manufacturer, is not guaranteed or endorsed by the publisher.
